# Reawakening of dormant estrogen-dependent human breast cancer cells by bone marrow stroma secretory senescence

**DOI:** 10.1186/s12964-018-0259-5

**Published:** 2018-08-17

**Authors:** Samir Tivari, Haiyan Lu, Tanya Dasgupta, Mariana S. De Lorenzo, Robert Wieder

**Affiliations:** 1 Department of Medicine, Rutgers New Jersey Medical School and Rutgers Cancer Institute of New Jersey, 205 South Orange Avenue, Cancer Center H1216, Newark, NJ 07103 USA; 2 Department of Cell Biology and Molecular Medicine, Rutgers New Jersey Medical School and Rutgers Cancer Institute of New Jersey, Newark, NJ USA

**Keywords:** Breast cancer, Dormancy, Bone marrow stroma, Secretory senescence, Epithelial mesenchymal transition

## Abstract

**Background:**

Dormant estrogen receptor positive (ER+) breast cancer micrometastases in the bone marrow survive adjuvant chemotherapy and recur stochastically for more than 20 years. We hypothesized that inflammatory cytokines produced by stromal injury can re-awaken dormant breast cancer cells.

**Methods:**

We used an established in vitro dormancy model of Michigan Cancer Foundation-7 (MCF-7) breast cancer cells incubated at clonogenic density on fibronectin-coated plates to determine the effects of inflammatory cytokines on reactivation of dormant ER+ breast cancer cells. We measured induction of a mesenchymal phenotype, motility and the capacity to re-enter dormancy. We induced secretory senescence in murine stromal monolayers by oxidation, hypoxia and estrogen deprivation with hydrogen peroxide (H_2_O_2_), carbonyl-cyanide m-chlorophenylhydrazzone (CCCP) and Fulvestrant (ICI 182780), respectively, and determined the effects on growth of co-cultivated breast cancer cells.

**Results:**

Exogenous recombinant human (rh) interleukin (IL)-6, IL-8 or transforming growth factor β1 (TGFβ1) induced regrowth of dormant MCF-7 cells on fibronectin-coated plates. Dormant cells had decreased expression of E-cadherin and estrogen receptor α (ERα) and increased expression of N-cadherin and SNAI2 (SLUG). Cytokine or TGFβ1 treatment of dormant clones induced formation of growing clones, a mesenchymal appearance, increased motility and an impaired capacity to re-enter dormancy. Stromal injury induced secretion of IL-6, IL-8, upregulated tumor necrosis factor alpha (TNFα), activated TGFβ and stimulated the growth of co-cultivated MCF-7 cells. MCF-7 cells induced secretion of IL-6 and IL-8 by stroma in co-culture.

**Conclusions:**

Dormant ER+ breast cancer cells have activated epithelial mesenchymal transition (EMT) gene expression programs and downregulated ERα but maintain a dormant epithelial phenotype. Stromal inflammation reactivates these cells, induces growth and a mesenchymal phenotype. Reactivated, growing cells have an impaired ability to re-enter dormancy. In turn, breast cancer cells co-cultured with stroma induce secretion of IL-6 and IL-8 by the stroma, creating a positive feedback loop.

## Background

Breast cancers metastasize early, as soon as cells with diminished cohesion enter the circulation through newly formed, leaky blood vessels [1, 2]. They undergo massive attrition, but some survive and settle in distant organs such as bone marrow, where their numbers are further reduced by a hostile environment. The ones that survive have tumor initiating capacity [3], become dormant and undetected for years [4] and are resistant to adjuvant chemotherapy administered with the very intent of eliminating them [5]. Dormancy occurs primarily in estrogen receptor positive (ER+) breast tumors [6]. Dormant cells continue to reactivate for decades in a stochastic manner, consistent with a microenvironment-dependent process. Most recur years after adjuvant antiestrogen therapy or menopause, suggesting that estrogen deprivation may adversely affect the microenvironment’s ability to support dormancy [7, 8].

Dormancy is considered as either tumor mass dormancy or cellular dormancy [9]. Tumor mass dormancy maintains a balance between proliferation and cell death through limiting blood supply and immune suppression. Cellular dormancy maintains cells at the single or oligocellular level by inhibiting cell division and promoting re-differentiation through interactions with the microenvironment. The latter may also apply to tumor mass dormancy [9]. Many mechanisms for inducing and escaping cellular dormancy have been identified, which vary with different metastatic microenvironments and the different origins of primary tumors [10].

Early and late primary tumors result in disseminated bone marrow metastases with differing gene expression signatures [11]. Macrophages in premalignant lesions may induce epithelial-mesenchymal transition (EMT) and metastasis [12] and micrometastases from late, hypoxic tumors are already preprogrammed to be resistant to therapy [13].

The bone marrow microenvironment induces tumor dormancy through multiple mechanisms and contains many types of stromal cells that interact with disseminated cancer cells [14]. Studies suggest that dormant micrometastases can usurp the hematopoietic stem cell niche [15, 16], but both endosteal osteoblasts and non-cycling endothelial niches support dormancy [17]. Redundant dormancy-inducing signals derive from high levels of all-*trans* retinoic acid (ATRA), transforming growth factor-β-2 (TGFβ)2, bone morphogenic protein (BMP)-7 and a hypoxic environment in the bone marrow [17]. Hypoxia induces glucose transporter-1 (GLUT1) and hypoxia-inducible factor 1-α (HIF1α), key dormancy genes nuclear receptor subfamily 2 group F member 1 (NR2F1), which is an orphan nuclear retinoid receptor, DEC2, a basic helix-loop-helix transcription repressor involved in circadian rhythm, cyclin dependent kinase (CDK) inhibitor p27^Kip1^ and chemoresistance in ER+ breast cancer cells [17]. Leukemia inhibitory factor (LIF) provides dormancy signals through signal transducer and activator of transcription protein-3 (STAT3) and suppressor of cytokine signaling 3 (SOCS3) [18]. Osteoblasts [19] and hypoxia [20] induce dormancy through AXL receptor tyrosine kinase (Axl) and its ligand growth arrest-specific 6 (GAS6) and increased TGFβ2 and its receptor [13]. ATRA also induces NR2F1 and TGFβ2 and mediates quiescence through transcription factor SOX9, retinoic acid receptor β (RARβ) and CDK inhibitors [21]. NR2F1 also acts through global chromatin repression and the pluripotency gene NANOG [21].

TGFβ2 induces dormancy through stress-activated mitogen-activated protein kinase p38 signaling, which upregulates dormancy-associated proteins DEC2 and p27^Kip1^ [22]. High ratios of activated p38/ERK induce p38-mediated survival and dormancy signaling through activating transcription factor (ATF)/Ras homolog enriched in brain (RHEB)/mammalian target of rapamycin (mTOR) [23] and dormancy-associated transcription factors DEC2, p27^Kip1^, p21^WAF1^ and NR2F1 [21, 22]. p38 can be activated by urokinase-type plasminogen activator (uPA), fibronectin and integrins [24, 25]. BMP-7, a TGFβ family member secreted by stromal cells, can also induce reversible dormancy through induction of p38 signaling and upregulation of the metastasis suppressor gene N-myc downregulated gene 1 (NDRG1) [26].

Relapse after years of dormancy remains a significant medical problem. In the perivascular niche, non-dividing endothelial cells promote dormancy through thrombospodin-1 but sprouting neovascular endothelial cell tips promote micrometastatic outgrowth through TGFβ1 and periostin [27]. Estrogen depletion, associated with tumor relapse [7, 8] induces Angiopoietin-2, which destabilizes endothelial cell-cell junctions by disrupting Tie2 receptor and increases tumor cell surface integrin β1 [28]. Estrogen depletion also induces secretion of interleukin-6 (IL-6) by metastatic cells in an autocrine manner through IL-6/Stat3/neurogenic locus notch homolog protein 3 (Notch3) and reactivation into a hormone resistant population [3]. Osteoclast activity induced by receptor activator of nuclear factor kappa-Β ligand (RANKL) can also release dormant endosteal breast cancer micrometastases through vascular cell adhesion molecule 1 (VCAM-1) expression on the cancer cells [29, 30]. Fibrosis and Colagen-1 induce dormant cell reawakening [31]. ER+ dormant breast cancer cells expressing lysyl oxidase homolog 2 (LOXL2) acquire stem-like characteristics that depend on epithelial mesenchymal programs to mediate exit from dormancy [32]. Epigenetic events in the dormant microenvironment may also dictate awakening from dormancy [33].

We demonstrated a role for fibroblast growth factor-2 (FGF-2), which is abundant on the marrow stroma extracellular matrix, in the dormancy of ER+ breast cancer cells [34]. FGF-2 induces G_1_ cell cycle arrest [35], partial re-differentiation of ER+ cells and re-expression of integrin α5β1, a receptor for fibronectin also abundant in the marrow [34]. FGF-2 and integrin α5β1 initiate survival signaling through phosphoinositol-3 kinase (PI3K) and microtubule-associated protein kinase (MAPK) [34, 36]. They inactivate the small guanosine triphosphatase (GTPase) RhoA through membrane localization of the Rho GTPase activating protein (GAP) GTPase Regulator Associated with the Focal Adhesion Kinase pp125(FAK) (GRAF) [37], resulting in cortical redistribution of fibrillar actin (F-actin) and a characteristic large, flat dormant cell phenotype with large cytoplasm to nucleus ratios.

Because of a required close association with stroma, we hypothesized that continued “health” of the bone marrow stroma is key to maintaining dormancy. Potential injury to stromal cells over many years, aging and estrogen deprivation during menopause may result in secretory senescence [38], characterized by production of inflammatory cytokines able to reawaken dormant cancer cells.

Several observations support this hypothesis. Constitutive secretion of interleukin (IL)-6 by cultured bone marrow stroma positively correlates with age, and aging predisposes cells to injury-induced secretory senescence [39]. Hormone replacement lowers secretion of IL-6 and IL-11 [39]. Declines in ATP levels induce apoptosis and promote necrotic inflammation [40] and hydrogen peroxide (H_2_O_2_) induces inflammatory cytokines (IL-6, IL-7, IL-16, and IL-17) and necrosis in aging fibroblasts. Aging also eliminates protection from hypoxic or oxidative stress by heat preconditioning-induced heat shock protein 70 (HSP70) in dermal fibroblasts [41] and decreases the HSP70 response and adaptation in rat livers [42].

In turn, senescent fibroblasts promote malignant transformation of co-cultivated premalignant epithelial cells [38]. Aging adipocytes increase cytokine secretion and stimulate tumor growth [43], events with potential impact as the ratio of fat to hematopoietic cells in the marrow increases with age [44]. Here, we test the hypothesis that oxidative, hypoxic and estrogen deprivation-induced bone marrow stromal injury promotes secretory senescence and that cytokines produced by the stroma can reactivate dormant ER+ breast cancer cells in an in vitro dormancy model.

## Methods

### Cells

Human luminal ERα-positive MCF-7 breast cancer cell line was obtained from American Type Culture Collection (ATCC) (Manassas, VA, USA) and cultured at 37 °C at 5% CO_2_ Dulbecco’s modifies Eagle’s medium (DMEM) supplemented with 10% fetal bovine serum (FBS) (Serum Source International, NC), 1% penicillin/streptomycin and 1% L-glutamine (Corning, Corning, NY) as before [35]. The cell line was authenticated by the Rutgers New Jersey Medical School Molecular Resource Facility and demonstrated a 93% match to the ATCC cell line HTB-22 (Human MCF-7 Breast Adenocarcinoma).

### Supplies and reagents

Tissue culture- and fibronectin-coated plates were purchased from Corning (Corning, NY). Recombinant human (rh)FGF-2, rhIL-6, rhIL-8 and rhTGFβ1 and mouse blocking monoclonal antibody to CXCL1/KC (IL-8) and mouse immunoglobulin G (IgG) were purchased from R&D Systems (Minneapolis, MN). H_2_O_2_, carbonyl-cyanide m-chlorophenylhydrazzone (CCCP) and Fulvestrant (ICI 182780) were purchased from Sigma-Aldrich (Saint Louis, MO). Radioimmunoprecipitation assay (RIPA) buffer was purchased from Santa Cruz Biotechnology (Santa Cruz, CA). Rabbit monoclonal antibodies to human E-Cadherin (clone 24E10), N-Cadherin (clone D4R1H) XP), Slug (clone C19G7), β-Catenin (clone D10A8) were purchased from Cell Signaling (Danvers, MA). Mouse monoclonal antibody to estrogen receptor α (ERα) (clone F-10), N-cadherin (clone H-4), horseradish peroxidase (HRP)-labeled goat anti-rabbit IgG and goat anti-mouse IgG were purchased from Santa Cruz Biotechnology (Santa Cruz, CA). Vybrant CM-DiI fluorescent tracking dye was purchased from Invitrogen-Molecular Probes (Carlsbad, CA).

### Primary bone marrow stroma (BMS)

Animal studies were approved by the Institutional Animal Care and Use Committee. Human bone marrow aspirates were obtained under an Institutional Review Board-approved protocol after informed consent. Bone marrow stroma was prepared from mouse and human bone marrow, as reported [34, 45]. Single cell suspensions of bone marrow hematopoietic progenitors, bone marrow flushed from mouse femurs and buffy coats from human aspirates were cultured in 25-cm^2^ flasks in Gartner’s Medium at 1.5 to 3 × 10^6^ cells/cm^2^ at 37 °C and 5% CO_2_. The medium and non-adherent cells were demi-depleted every 7 days and replaced with fresh medium until adherent stroma reached approximately 50–75% confluence. Stroma were detached by trypsin treatment, distributed into 24-well tissue culture-coated plates at 1–1.5 × 10^5^ cells/cm^2^ and cultured to confluence.

### Co-cultivation studies

MCF-7 breast cancer cells were labeled with Vybrant CM-DiI as per manufacturer’s instructions and their clonogenic efficiency for forming growing and dormant clones on fibronectin-coated tissue culture plates was confirmed to be identical to that of unlabeled cells. Prior to co-incubation, confluent stromal monolayers on quadruplicate wells of 24 well plates were treated with variable concentrations of H_2_O_2_ and CCCP in fetal calf serum-free DMEM or with DMEM alone as control for one hour, washed twice with DMEM/10% FCS and incubated with one thousand Vybrant CM-Dil-labeled MCF-7 cells in one ml DMEM/10% FCS for six days at 37 °C 5% CO_2_. For experiments designed to inhibit estrogen signaling, the Gartner’s medium was aspirated from confluent stroma wells and replaced with 1000 MCF-7 cells in either 1 ml phenol red-free DMEM/10% FCS with ICI 182780 at various concentrations or in 1 ml phenol red-free DMEM/10% FCS as control, and were incubated for 6 days. For antibody blocking experiments, 2 μg/ml blocking monoclonal antibody to CXCL1/KC or mouse IgG was included with the MCF-7 cells at the time of incubation. Day 6 embedded colonies and detached spherules were photographed using a Zeiss Axio Observer Z1 Microscope equipped with Zeiss Axiocam MRm Camera (Zeiss, Germany) and counted.

### Breast cancer Clonogenic assays on fibronectin-coated tissue culture plates

MCF-7 cells were incubated at clonogenic density with 1000 or 1500 cells/well in quadruplicate on human fibronectin coated 24 well plates in 1 ml DMEM/10% FCS and allowed to adhere overnight [34, 46]. The next day, wells were supplemented with 100 μl of 100 ng/ml of FGF-2 for a final concentration of 10 ng/ml with or without 10 ng/ml recombinant human IL-6, IL-8 or TGFβ1. After 6 days, cells were stained with 0.1% crystal violet and growing colonies of ≥ 30 cells and dormant colonies of 2–12 cells were counted. In experiments testing the effects of cytokines on dormant cells, fresh medium or medium containing 10 ng/ml recombinant human IL-6, IL-8 or TGFβ1 were added to the day 6 cultures. After an additional 6 days, growing and dormant colonies were counted after fixing in crystal violet.

### Western blot analysis

Cell lysates were prepared as before, analyzed by sodium dodecyl sulfate polyacrylamide gel electrophoresis (SDS-PAGE) and transferred to polyvinylpyrolidone membranes [35]. Indirect immunofluorescence was detected by enhanced chemiluminescence (ECL) and equal loading was confirmed by stable abundant protein bands on Coomassie Blue-stained membranes.

### Enzyme-linked immunoassay (ELISA

Mouse and human IL-6 and IL-8 concentrations in murine and human bone marrow stroma conditioned medium were carried out using Mouse IL-6 and Human IL-6 Elisa Kits (BD Biosciences, Franklin Lakes. NJ) and Quantikine Mouse CXCL1/KC and Quantikine Human CXCL8/IL-8 ELISA kits (R&D Systems, Minneapolis, MN). DMEM/10% FCS was collected from nearly confluent mouse and human stromal monolayers 24, 48 and 72 h after H_2_O_2_ and CCCP injury and from control plates or after 24 and 48 h incubations with ICI 182780 in phenol red-free medium and controls in triplicate in 24 well tissue culture plates. All experiments were done twice and most were done three times. Absolute values of cytokine secretion varied between experiments due to variable length of time needed to generate stromal cultures from mouse bone marrow. However, experimental trends remained consistent from experiment to experiment.

### Cell migration

Cell migration experiments were performed using ACEA xCELLigence system CIM-plates, as described previously [47], according to the manufacturer’s instructions. The migration of single cell suspensions of 1 × 10^5^ growing MCF-7 cells and growing MCF-7 cells treated with rhIL-6, IL-8 or TGFβ1 for 6 days and of dormant MCF-7 cells (MCF-7 cells treated with FGF-2 for 6 days) and dormant MCF-7 cells treated with rhIL-6, IL-8 or TGFβ1 for an additional 6 days was determined in triplicate. Each experiment was conducted at least twice.

### Live cell time lapse imaging

A time-lapse experiment was performed to image regrowth of individual growing and dormant colonies using a fully motorized Zeiss Axio Observer Z1 Microscope equipped with Zeiss Axiocam MRm Camera (Zeiss, Germany). MCF cells were incubated at 4750 cells/well on fibronectin-coated 6 well plates without and with FGF-2 10 ng/ml. Day 6 growing clones and dormant clones generated by 6 day incubations with FGF-2 were incubated with fresh DMEM/10% FCS without or with 10 ng/ml rhIL-6, rhIL-8 or rhTGFβ1 on 6-well fibronectin-coated plates and imaged every 24 h for 6 additional days. Growing and cytokine-treated colonies were photographed daily by an automated software control using the ZEN imaging software program, which includes a Tiles tool (Zeiss, Germany), permitting time course imaging of individual clones over a large area with the option to zoom-in on regions of interest.

### Statistical analysis

Statistical analyses were performed in Microsoft Excel using student unpaired *t*-test. A *p*-value less than 0.05 was considered statistically significant. Data are presented as mean ± standard deviations. All experiments were repeated a minimum of two times.

## Results

### Reactivation of dormant MCF-7 colonies in an in vitro dormancy model

Our dormancy model involves incubation of ER+ cells at clonogenic density, where the primary interaction of the cells is with the substratum instead of each other, on fibronectin-coated tissue culture plates in the presence of FGF-2 for six days [34]. In response to FGF-2, cells exit the cell cycle, partially re-differentiate, re-express integrins lost with de-differentiation, take on a spread-out appearance with omnidirectional focal adhesion complex activation and cortical redistribution of actin and form colonies of 2–12 cells. They re-express integrin α5β1, which binds fibronectin in the substratum and contributes to the FGF-2-mediated survival and re-differentiation signaling that results in the dormant phenotype [34, 36, 37].

**Fig. 1:**
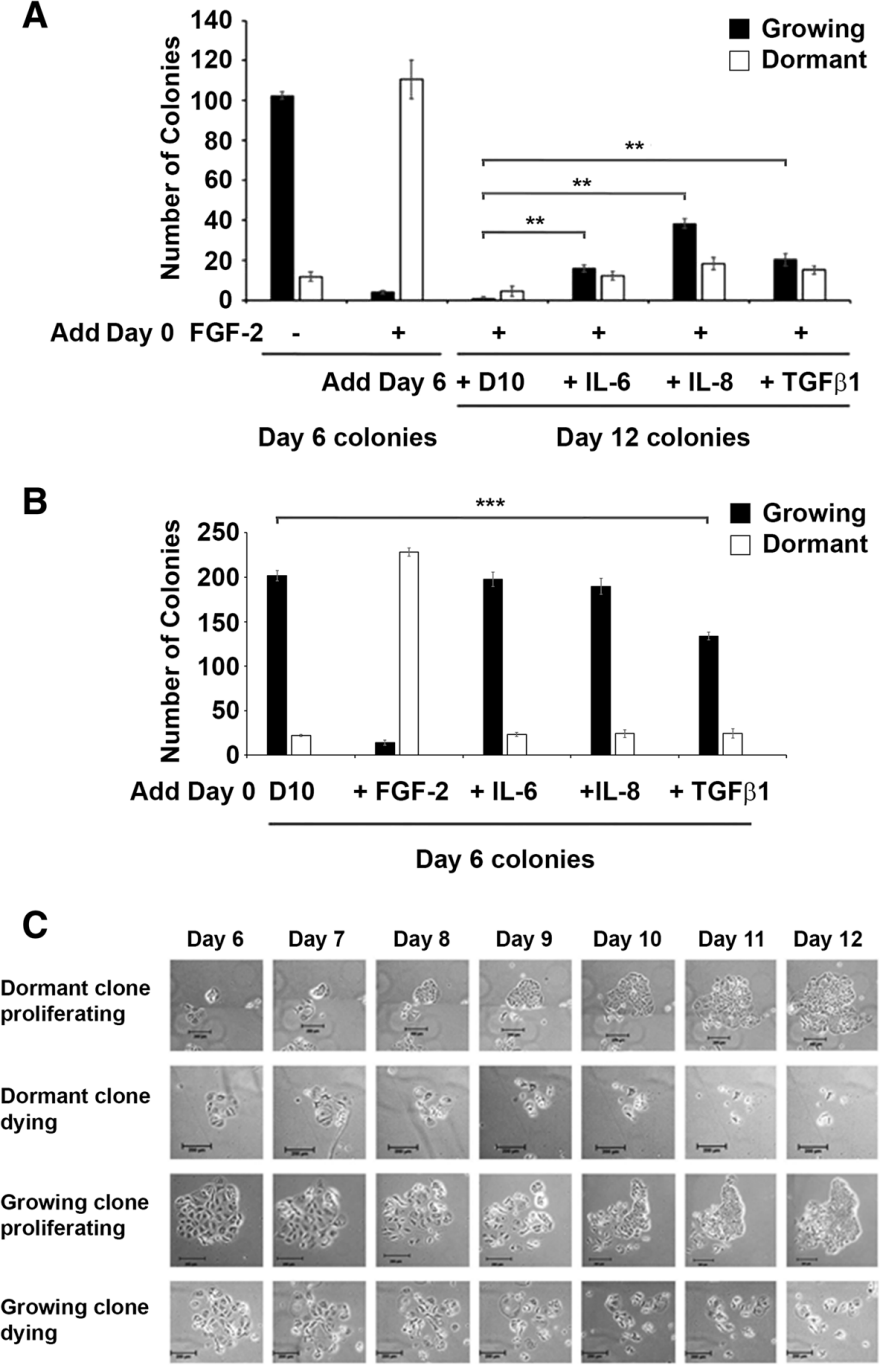
Exogenous recombinant human IL-6, IL-8 and TGFβ1 induce reactivation of dormant breast cancer colonies but do not promote growth of growing colonies. **a** Cytokines induce regrowth of dormant colonies. One thousand MCF-7 cells were incubated in fibronectin-coated wells with FGF-2 for 6 days, followed by addition of recombinant human IL-6, IL-8 and TGFβ1 on day 6 and incubation for an additional 6 days, as described. Cells were stained with 0.1% crystal violet and growing and dormant colonies were counted on day 6 and 12. **b** Cytokines do not promote the growth of growing colonies. MCF-7 cells were incubated in fibronectin-coated wells with FGF-2 or recombinant human IL-6, IL-8 or TGFβ1, as described. Cells were stained with 0.1% crystal violet and growing and dormant colonies were counted on day 6. **c** Time lapse photography demonstrating regrowth potential of dormant clones. Photographs demonstrate growth expansion or degradation of both dormant and growing colonies. MCF cells were incubated on fibronectin-coated plates without and with FGF-2 for six days, followed by an additional 6 day incubation with fresh DMEM/10% FCS without or with rhIL-6, IL-8 and TGFβ1, as described. Time-lapse microscopic images of the same individual colonies in the same fields were obtained once daily for these six days. Size markers are 200 μM

We tested the hypothesis that IL-6, IL-8 and TGFβ1 could reactivate dormant MCF-7 cells in this well-characterized in vitro model [34, 46]. Following our standard procedures, we incubated MCF-7 cells at clonogenic density on fibronectin-coated plates with rhFGF-2 for six days and observed the formation of mainly dormant clones, consistent with prior results (Fig. 1a). When these cultures were re-incubated with fresh medium without rhFGF-2, or fresh medium without rhFGF-2 but with added rhIL-6, rhIL-8 or rhTGFβ1 for an additional 6 days, the number of dormant clones was significantly diminished compared to the number on day 6. Instead, the number of growing colonies in cultures incubated with the cytokines or TGFβ1 was significantly higher than those in the cultures with medium alone (Fig. 1a). In contrast to the effects of the cytokines and rhTGFβ1 on dormant clones, rhIL-6, rhIL-8 or rhTGFβ1 did not enhance the growth of growing colonies. However, rhTGFβ1 did inhibit their formation, in line with our prior observations [48] (Fig. 1b).

Because a small number of growing clones do develop in day 6 rhFGF-2-treated dormant cultures, we followed the fate of single clones to demonstrate unequivocally the capacity of individual dormant clones to reactivate in response to cytokines and TGFβ1 through time lapse photography using a motorized Zeiss Axio Observer Z1 Microscope. Fig. 1c demonstrates that individual dormant clones are capable of being reactivated into growing clones. The observations also demonstrated that some dormant clones die, some growing clones die and occasional growing clones continue to proliferate.

**Fig. 2:**
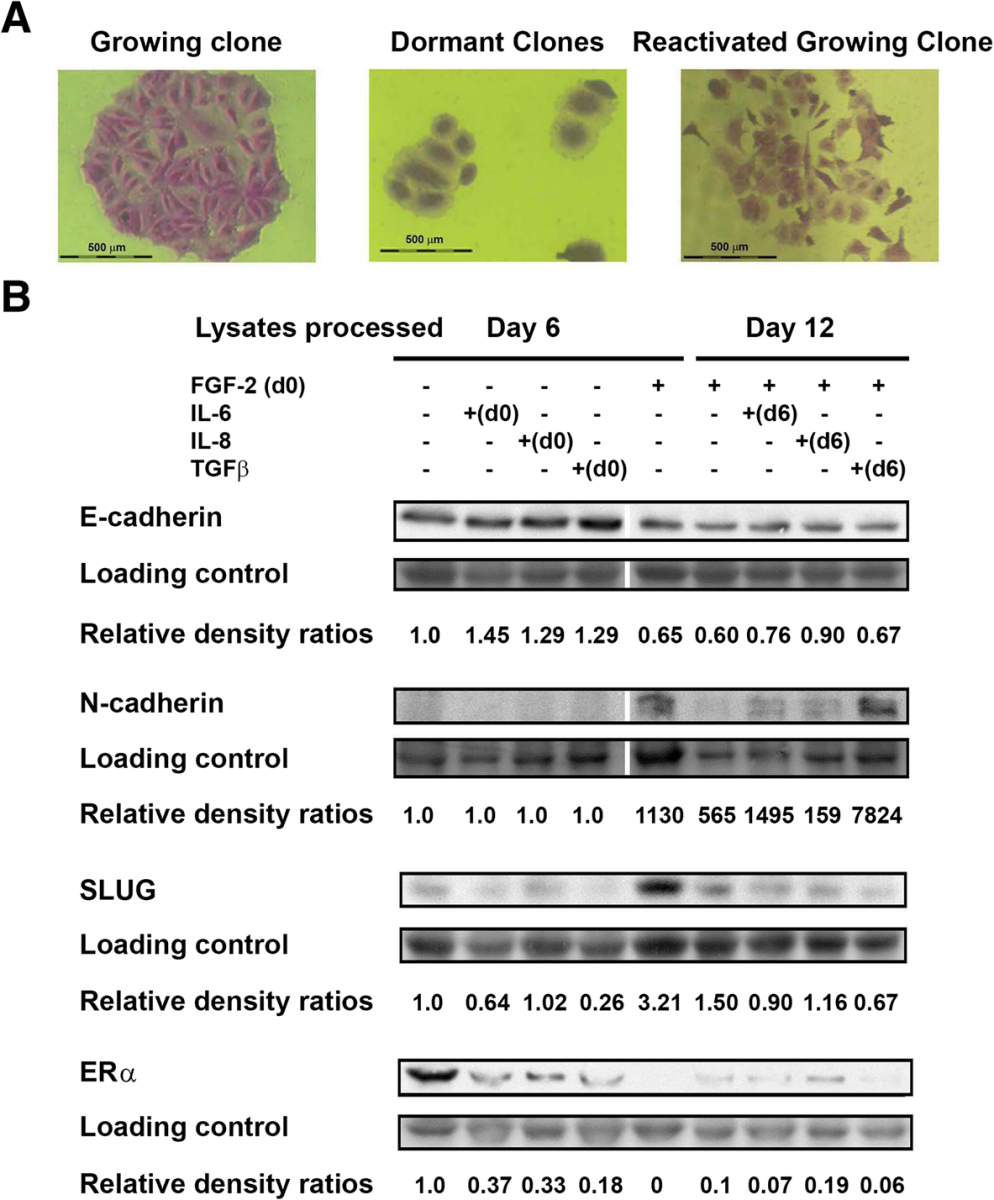
Dormant and reactivated dormant breast cancer colonies exhibit characteristics of an epithelial to mesenchymal transition. **a** Morphological changes from epithelial (left) to a spindle like (right) morphology were observed between growing MCF-7 colonies and reactivated dormant cells. Colonies were photographed at 40X magnification. **b** Western blots performed with lysates from MCF-7 cells incubated with or without FGF-2 in tissue culture for 6 days and dormant cells incubated with rhIL-6, IL-8 and TGFβ1 for an additional 6 days on fibronectin at clonogenic density. Blots were stained with antibodies to human E-cadherin, N-cadherin, SLUG, and ERα. Coomassie blue-stained gels were used as loading controls. Relative band intensity ratios to those of the corresponding loading control were determined

The appearance of many of the cells in the growing colonies that arose from dormant clones was different than that of the cells in the growing clones that formed from initial incubation of growing MCF-7 cultures for 6 days (Fig. 2a). Many of the reactivated cells appeared fusiform, reminiscent of fibroblasts, and had less inter-cell cohesion. This suggested that some of the reactivated dormant MCF-7 cells may have undergone EMT. We tested this hypothesis. To our surprise, the Western blots demonstrated that before reactivation, dormant cells have already activated their EMT genetic program by downregulation of E-cadherin and upregulation of N-cadherin and SLUG (Fig. 2b). Treatment with cytokines also caused a downregulated expression of ERα in growing MCF-7 cells and dormant cells stopped expressing it altogether. Dormant cells treated with cytokines and TGFβ1 also expressed very low levels of ERα.

**Fig. 3:**
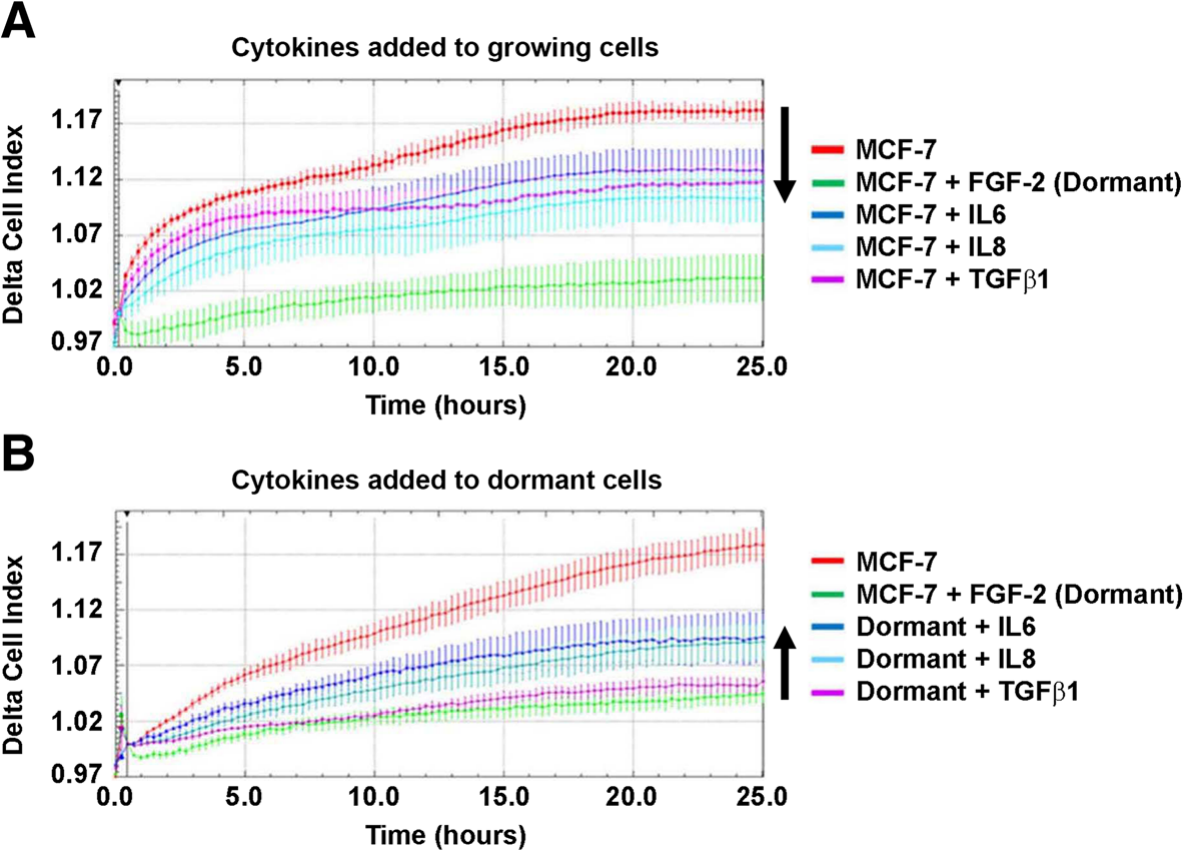
Inflammatory cytokines have opposite effects on the migratory properties of growing and dormant cells. **a** Cytokines decrease the motility of growing cells. Fifty thousand MCF-7 cells were incubated on fibronectin-coated 10 cm plates. The following day (day 0), DMEM/10% FCS without or with recombinant human FGF-2, IL-6, IL-8 or TGFβ1 were added to the plates to achieve a final concentration of 10 ng/ml. On day 6, cells were detached into single cell suspension by trypsin and 100,000 cells were added to the CIM-plate wells. Arrow indicates decreased motility of cytokine-treated growing cells compared to that of growing cells. **b** Cytokines increase the motility of dormant cells. Fifty thousand MCF-7 cells were incubated on fibronectin-coated 10 cm plates and DMEM/10% FCS and FGF-2 was added the following day. Six days later, DMEM/10% FCS without or with recombinant human IL-6, IL-8 or TGFβ1 were added to the cultures. On day 12, cells were detached into single cell suspension by trypsin and 100,000 cells were added to each well of the CIM-plate. Trypsin-dispersed day 6 dormant cells were used as negative controls. Arrow indicates increased motility of cytokine-treated dormant cells compared to that of dormant cells. Results represent the average of triplicate wells

In addition to the phenotypic observations shown, incubation of growing cells with rhIL6, rhIL-8 and rhTGFβ1 slowed their motility (Fig. 3a), while incubation of dormant cells with rhIL-6 and rhIL8 increased motility (Fig. 3b). In the experiment shown, TGFβ1 had only a small effect on promotion of dormant MCF-7 cell motility but had a greater effect in other replicate experiments.

### Dormant cells or cells reactivated from dormancy have a decreased capacity to re-enter dormancy

**Fig. 4:**
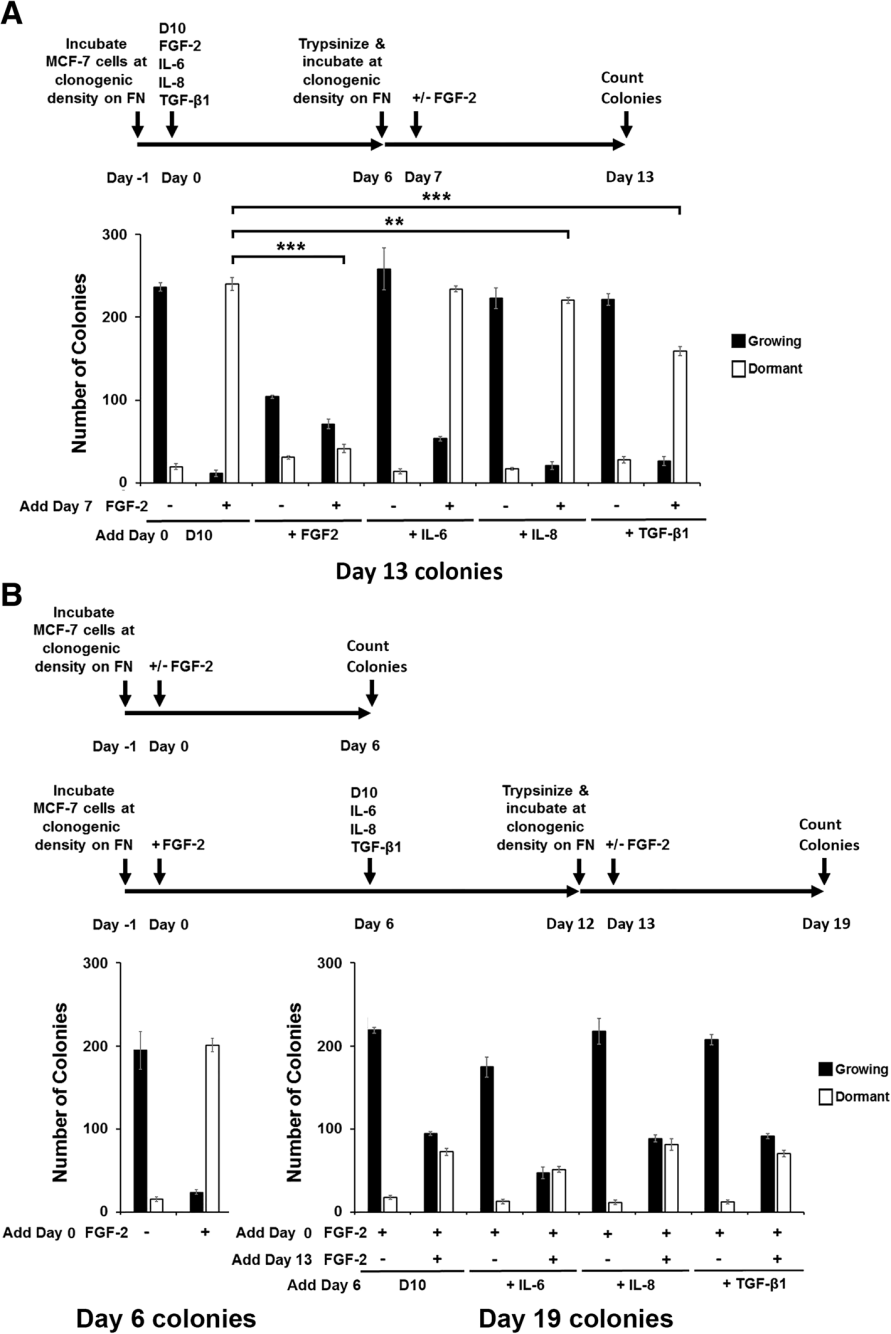
Dormant and reactivated dormant colonies have an impaired ability to re-enter the dormant state. **a** A total of 50,000 MCF-7 cells were incubated on fibronectin-coated 10 cm plates. The following day (day 0) DMEM/10% FCS without or with recombinant human FGF-2, IL-6, IL-8 or TGFβ1 were added to the wells to achieve a final concentration of 10 ng/ml. On day 6, cells were detached into single cell suspension by trypsin and 1000 cells/well were incubated in quadruplicate on 24 well fibronectin-coated plates. On day 7 DMEM/10% FCS without or with FGF-2 at a final concentration of 10 ng/ml was added to the cultures. On day 13, wells were stained with 0.1% crystal violet and growing and dormant colonies were counted. **b** MCF-7 cells were incubated on fibronectin-coated 10 cm plates. The following day (day 0) DMEM/10% FCS with FGF-2 was added to the wells to achieve a final concentration of 10 ng/ml. On day 6, DMEM/10% FCS without or with recombinant human IL-6, IL-8 or TGF-β1 was added to the wells to achieve a final concentration of 10 ng/ml. On day 12, cells were detached into single cell suspension by trypsin and 1000 cells/well were incubated in quadruplicate on 24 well fibronectin-coated plates. On day 13, DMEM/10% FCS without or with FGF-2 at a final concentration of 10 ng/ml was added to the cultures. On day 19, wells were stained with 0.1% crystal violet and growing and dormant colonies were counted. In a control experiment, MCF-7 cells were incubated on fibronectin-coated 24-well plates at 1000 cells/well. DMEM/10% FCS without or with FGF-2 at a final concentration of 10 ng/ml was added the next day. Six days later, wells were stained with 0.1% crystal violet and growing and dormant colonies were counted. Results represent the average of quadruplicate wells. Error bars: Standard Deviation. *p < 0.05, **p < 0.01 ***p < 0.001

The evidence that dormant cells have already activated their EMT program led us to explore the capacity of dormant cells or dormant cells reactivated with inflammatory cytokines or rhTGFβ1 to form dormant clones again. Figure 4a demonstrates that dormant clones that were disbursed by trypsinization and reincubated as single cells at clonogenic density have a significantly diminished capacity to form dormant clones. Parenthetically, their growing colony clonogenic potential is also diminished. Growing clones treated with rhIL-6 for 6 days have no change in their growing or their dormant clonogenic potential. Growing clones treated with rhIL-8 or rhTGFβ1 on the other hand, have a small but significantly impaired dormant clonogenic potential. To determine the effects of rhIL-6, rhIL-8 and rhTGFβ1 treatment of dormant clones on their ability to re-form dormant clones, we treated dormant clones for 6 days with rhIL-6, rhIL-8 and rhTGFβ1, disrupted clones with trypsin to generate single cell suspensions and re-incubated the cells at clonogenic density on fibronectin with and without rhFGF-2. Figure 4b demonstrates that dormant clones that were reactivated with either DMEM/10% FCS (D10), rhIL-6, rhIL-8 or rhTGFβ1 had a very low capacity to form dormant clones, with growing to dormant clone ratios greater than 1 when incubated with FGF-2. These data demonstrate that cells in dormant clones, which have already undergone EMT, have a significantly impaired capacity to re-enter FGF-2-induced dormancy once activated to grow again in our in vitro model.

### Stromal injury results in secretion of inflammatory cytokines and activation of TGFβ and TNFα

**Fig. 5:**
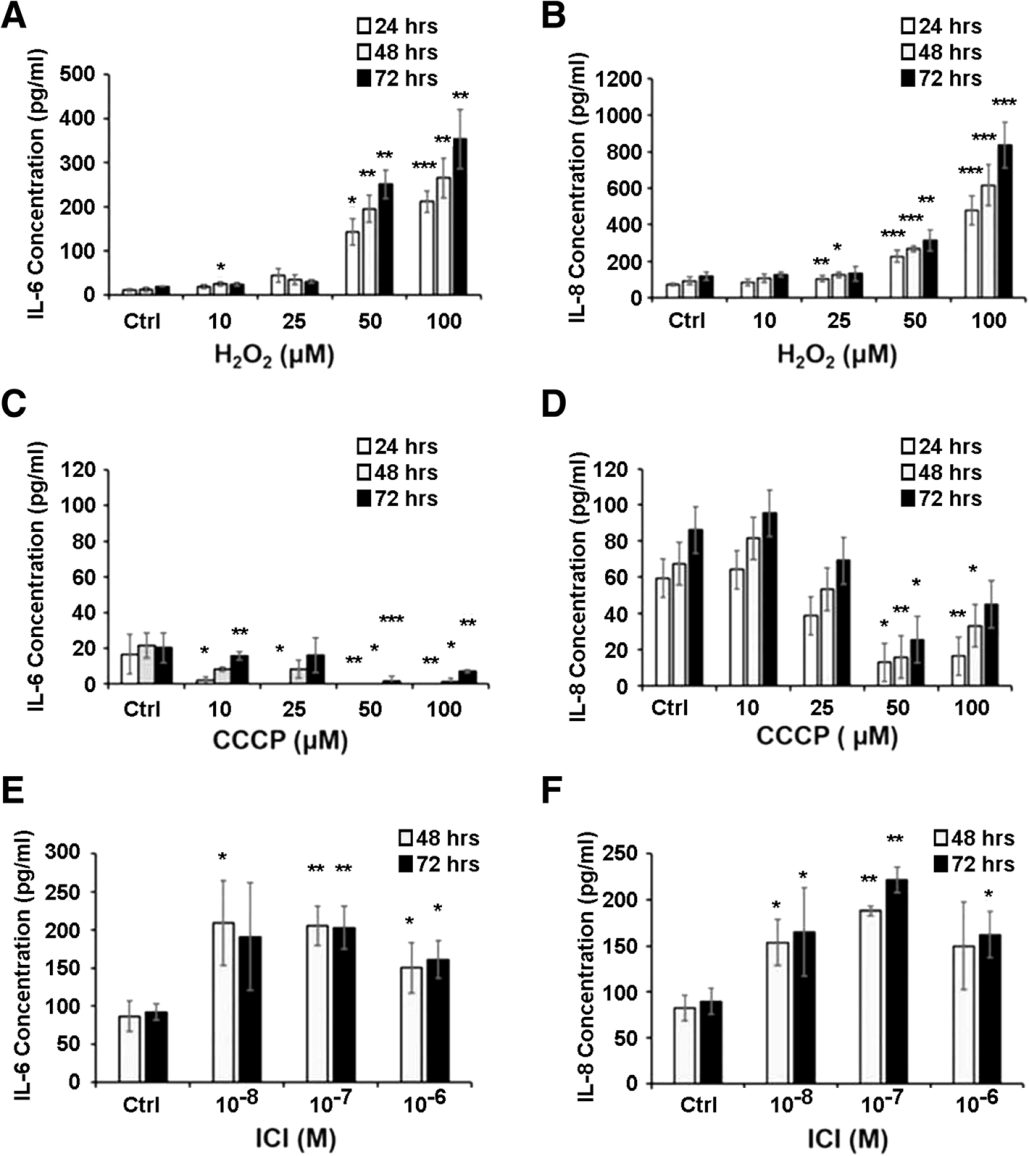
Oxidative stress and estrogen deprivation promote higher production of IL-6 and IL-8 secretion in murine bone marrow stromal cells. ELISA assays demonstrated significantly higher levels of production of IL-6 (**a** and **e**) and IL-8 (**b** and **f**) in the conditioned medium from murine stromal layers treated with H_2_O_2_ and ICI 182780. Induction of hypoxia by CCCP did not cause similar effects (**c** and **d**). Near confluent stromal monolayers cultured in 24-well plates were treated with H_2_O_2_ and CCCP in serum-free medium for 1 h, washed once and incubated in DMEM/10%FCS medium. Supernatant samples were collected at 24, 48 and 72 h. Confluent stromal monolayers cultured in 24-well plates were treated with ICI 182780 in medium without phenol-red for 48 h and 72 h, washed once and incubated with DMEM/10%FCS medium. The IL-6 and IL-8 levels were determined using murine IL-6 and CXCL1/KC ELISA kit according to manufacturer’s instructions. Error bars: Standard Deviation. *p < 0.05, **p < 0.01 ***p < 0.001

**Fig. 6:**
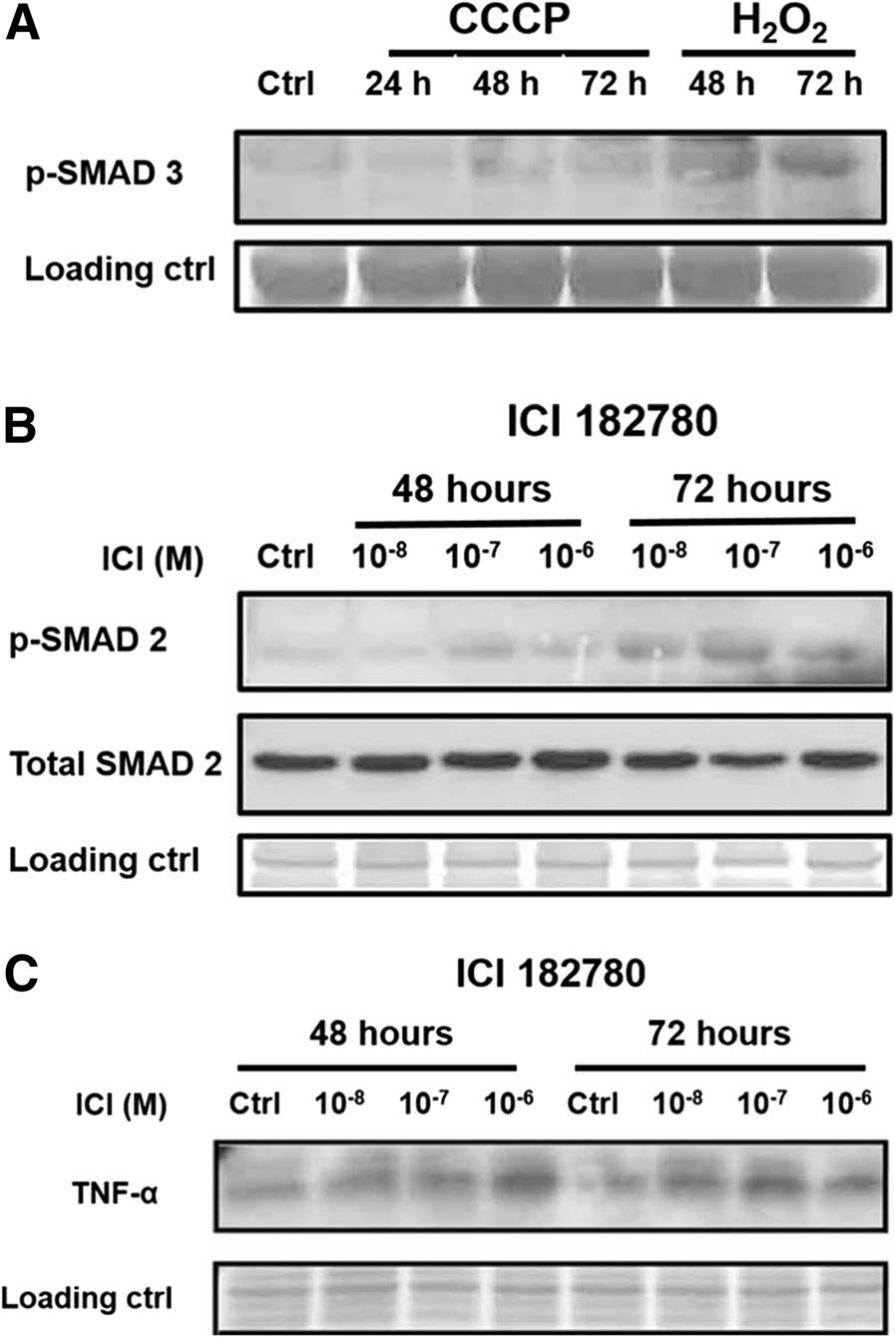
Oxidative stress, hypoxia and estrogen deprivation induces activation of TGFβ and TNFα signaling pathway. Western blots for (**a**). phospho-SMAD-3 of murine stromal cells treated with H_2_O_2_ and CCCP, and (**b**). phospho-SMAD-2 and (**c**). TNFα of mouse stromal cells treated with ICI 182780. Nearly confluent mouse stromal monolayers maintained in 10 cm plastic dishes were treated with 100 μM of H_2_O_2_ and CCCP for one hour, washed with PBS and incubated in DMEM/10%FCS for 24, 48, or 72 h. Mouse stromal monolayers were also treated with ICI 182780 at different concentrations (10^− 8^, 10^− 7^ and 10^− 6^ M) for 48 and 72 h. Cells were lysed in modified RIPA buffer and analyzed by SDS-PAGE with antibody to mouse phospho-SMAD-2, total SMAD-2, phospho-SMAD-3 and TNFα

We tested the hypothesis that injury of bone marrow stroma by oxidation, hypoxia or estrogen deprivation could induce secretory senescence. Exposure of human stromal monolayers obtained from several normal volunteers with H_2_O_2_, CCCP or ICI 182780 resulted in highly variable induction of IL-6 and IL-8 secretion and was found not to be a useful experimental model for our studies. We moved to a biologically less variable model with stroma from NCr nu/nu mice. Our results demonstrated that one hour incubations with H_2_O_2_ at concentrations at or above 50 μm (Fig. 5a and b) and 24 and 48 h incubations with ICI 182780 at or above 10^− 8^ M (Fig. 5e and f) consistently induced secretion of IL-6 and IL-8 by murine stroma after 24 h. Induction of hypoxia by poisoning the mitochondrial electron transport chain with CDDP for one hour did not induce secretion of IL-6 or IL-8 and, in fact, had the opposite effect (Fig. 5c and d). Injury to murine stroma also caused activation of the TGFβ signaling, as demonstrated by phosphorylation of SMAD-2 and SMAD-3 and estrogen deprivation induced activation of TNF-α (Fig. 6). As a result of these studies, we proceeded to conduct co-cultivation experiments with murine stroma.

### Stromal injury results in increased growth of co-cultured breast cancer colonies

**Fig. 7:**
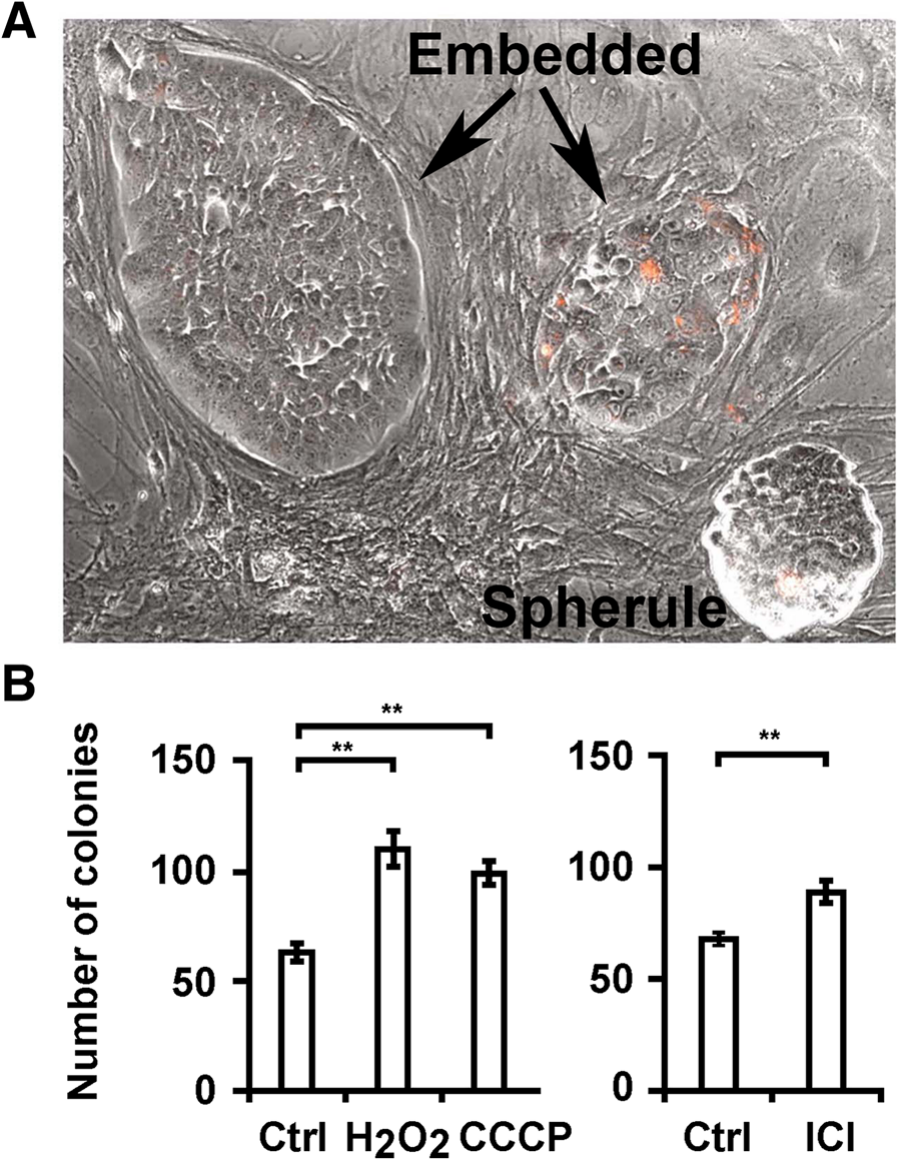
Stromal oxidation-, hypoxia-, and estrogen deprivation–induced injury permit outgrowth of co-cultivated breast cancer colonies. **a** Photomicrograph (100×) of two embedded breast cancer cell colonies (Embedded) and a non-adherent breast cancer cell spherule (Spherule) on murine bone marrow stroma monolayers after six days of incubation. Breast cancer cells were labeled with Vybrant CM-DiI prior to co-culture. **b** The number of embedded colonies after six days of co-incubation with stroma treated with control DMEM/10% FCS, DMEM/10% FCS with H_2_O_2_ (5 × 10^− 5^ M) and CCCP (5 × 10^− 5^ M) or stroma treated with ICI 182780 (10^− 7^ M) in phenol red-free DMEM/10% FCS or phenol-red-free control. Error bars: Standard Deviation. **p < 0.01

Confluent murine stromal monolayers in quadruplicate wells on 24 well plates were treated with H_2_O_2_ or CCCP for an hour or with ICI 182780 for two days, as described above. The medium was replaced with 1 ml DMEM/10% FCS containing 1000 Vybrant CM-DiI fluorescent dye tracker-labeled MCF-7 cells and the plates were re-incubated for 6 days. On day 6, colonies were counted and photographed. Figure 7a demonstrates the appearance of two types of colonies, ones that were flat and embedded in the 2-dimensional stroma monolayer consisting of several dozen cells and ones that were growing as three dimensional spheroids atop the monolayer. Embedded colonies on stroma injured with H_2_O_2_, CCCP or ICI 182780 were more frequent (Fig. 7b) after 6 days than colonies in control wells**.** The embedded colonies appeared to have dormant cells at the periphery but contained growing cells internally. It was apparent that cells internal to the colony escaped factors in mouse stroma sustaining human breast cancer cell dormancy. The frequency of the three dimensional spherules were not statistically different with stromal injury. These data suggest that the growth of embedded colonies was stromal injury-dependent but that of anchorage-independent spheroids was autonomous. Blocking experiments with anti-murine CXCL1/KC (IL-8) antibodies did not prevent increased numbers of the embedded 2-D colonies or the 3-D spherules. Blocking of IL-8 was selected because murine IL-8 binds human IL-8 receptors while murine IL-6 does not. Clearly, other factors from injured stroma also contributed to promotion of growth.

### Co-cultivation of stroma with MCF-7 cells induces secretion of IL-6 and IL-8

**Fig. 8:**
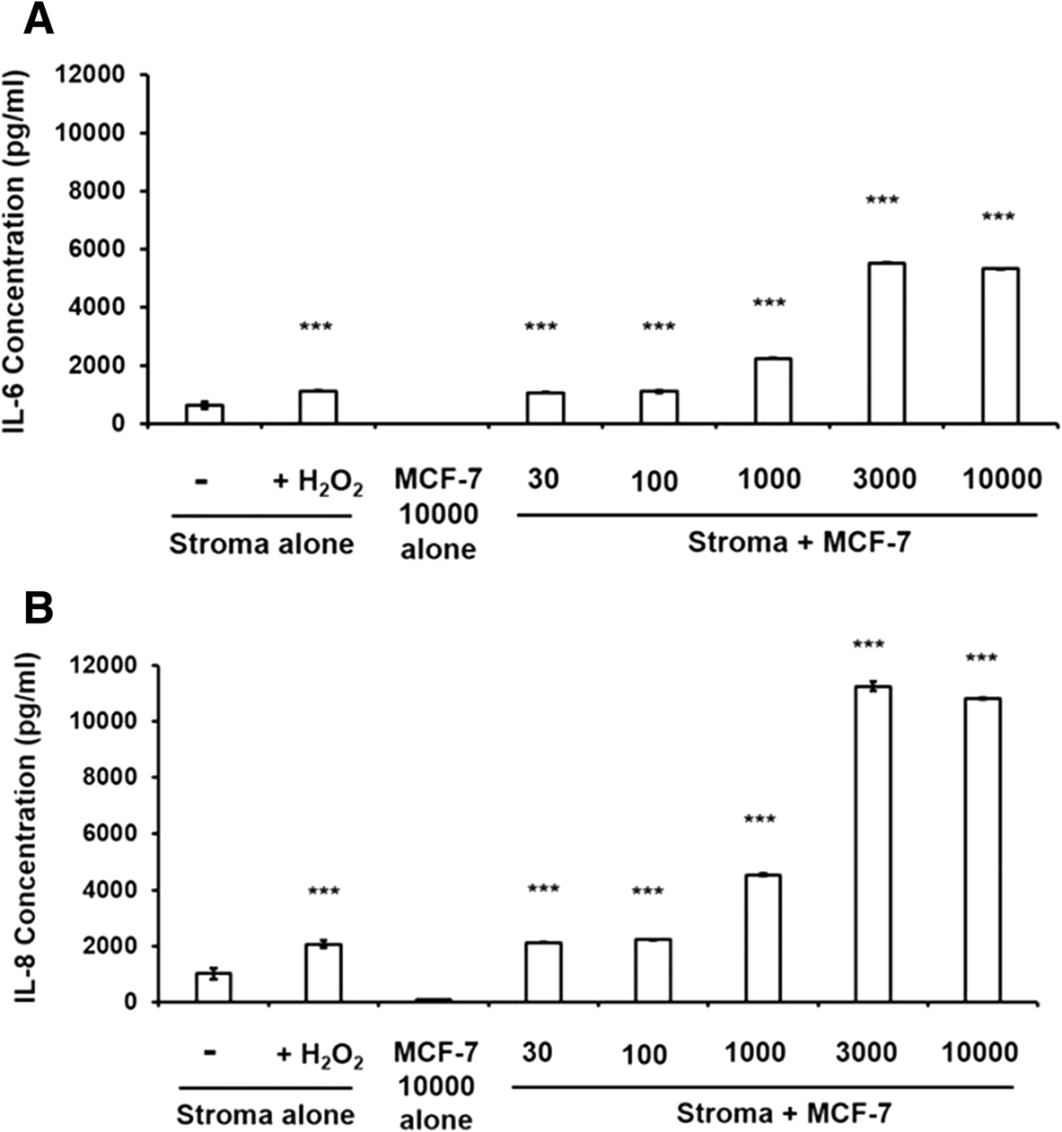
Co-cultivation of murine bone marrow stromal cells with MCF-7 breast cancer cells induces increases in secretion of IL-6 and IL-8. Confluent stroma were treated with H_2_O_2_ 100 μM for an hour, washed once with PBS, medium was changed to DMEM/5%FCS and conditioned medium was sampled for injury-induced IL-6 and IL-8 production as positive controls. Simultaneously, medium on uninjured stroma in triplicate wells were changed to DMEM/5%FCS containing variable numbers of MCF-7 cells and incubated for 48 h. Conditioned medium samples were collected 48 h after incubation and (**a**). IL-6 and (**b**). IL-8 concentrations were determined using murine IL-6 and CXCL1/KC ELISA kits, respectively, according to manufacturer’s instructions. Error bars: Standard Deviations. *p < 0.05, **p < 0.01 ***p < 0.001

**Fig. 9:**
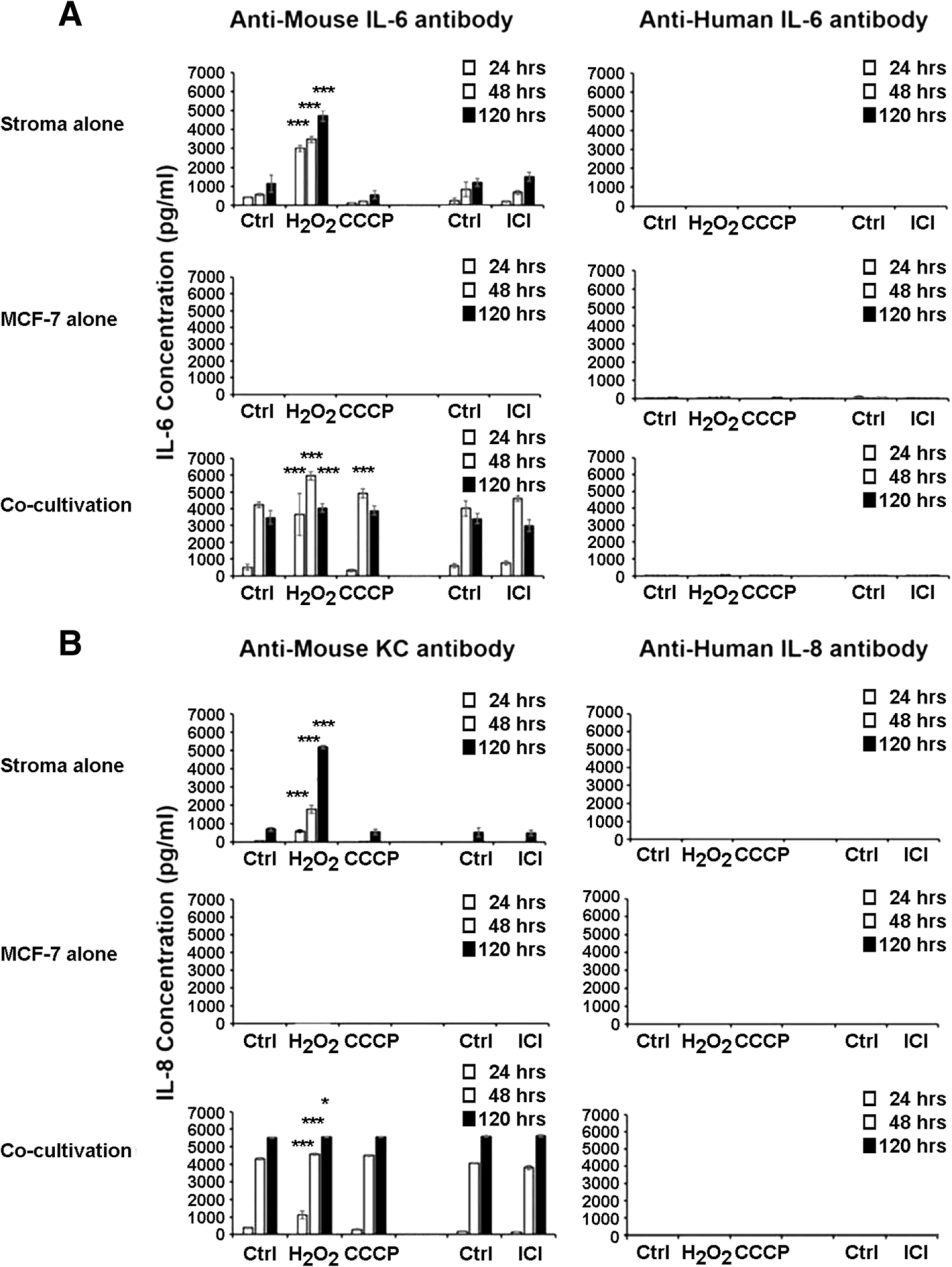
Bone marrow stromal cells are the primary contributors of production of IL-6 and IL-8 in co-cultivation with MCF-7 breast cancer cells. ELISA assays demonstrated significantly higher levels of production of murine (**a**). IL-6 and (**b**). IL-8. MCF-7 breast cancer cells (1000 cells/well) were seeded on human fibronectin coated 24-well plate. On day 3, cells were treated with H_2_O_2_ and CCCP for one hour in serum-free medium, and with ICI for 2 days in DMEM/10%FCS medium without phenol-red, washed once and incubated in DMEM/10%FCS. Confluent stromal monolayers were prepared from murine bone marrow stroma as before. At confluence, stromal monolayers were treated with H_2_O_2_ and CCCP for one hour and with ICI for 2 days, washed once and incubated in DMEM/10%FCS medium. MCF-7 breast cancer cells (1000 cells/well) were co-incubated with control and H_2_O_2_ 50 μM, CCCP 50 μM and ICI-treated stromal monolayers for 6 days. Supernatant samples were collected 24, 48 and 120 h and murine and human IL-6 and IL-8 levels in the supernatant were determined using murine and human IL-6 ELISA kit, and murine CXCL1/KC and human CXCL8/IL-8 ELISA kit according to the manufacturer’s instructions. Error bars: Standard Deviation. *p < 0.05, **p < 0.01 ***p < 0.001

We tested the hypothesis that breast cancer cells induced to proliferate by cytokines secreted by injured stroma may in turn feedback stimulate the stroma to sustain the secretory senescence initiated by injury. We co-incubated breast cancer cells with murine stroma and measured cytokine secretion in the conditioned media. Figure 8 demonstrates that co-incubation of breast cancer cells with murine stroma induced significant secretion of IL-6 and IL-8 into the conditioned medium in a dose dependent manner up to a maximum limit which was significantly higher than that achieved by H_2_O_2_ injury, used as a positive control. Similar data were obtained with human stroma. Control experiments measuring conditioned medium from chemically-injured murine stroma, from human breast cancer cells and from the two cell types cultured together using both murine and human cytokine antibodies demonstrates that IL-6 and IL-8 were produced almost entirely by the stromal cells when co-cultured with breast cancer cells (Fig. 9), suggesting that cancer cells induce further feedback injury to the stroma as they proliferate.

## Discussion

**Fig. 10:**
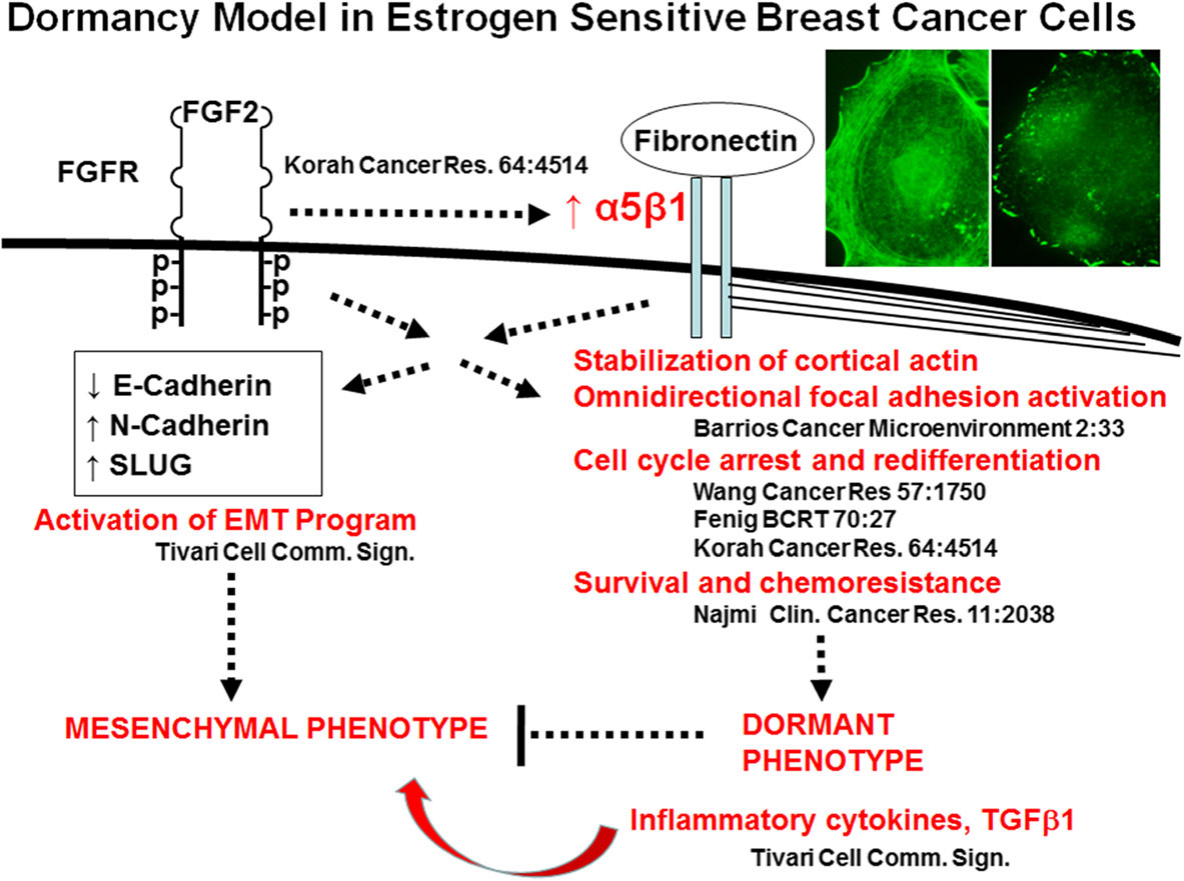
Schematic of the in vitro FGF-2-initiated dormancy model of estrogen sensitive human breast cancer cells. According to the model, FGF-2 induces re-expression of integrin α5β1 and together with its fibronectin-binding, a cell dormancy phenotype through cell spreading, cortical redistribution of fibrillar actin and omnidirectional activation of FAK, cell cycle arrest, survival and chemoresistance. FGF-2 and α5β1 also induce activation of the EMT gene expression program which can lead to a mesenchymal phenotype, but the transition is repressed by the dormancy program. This repression is released by inflammatory cytokines and TGFβ1, outlined within this study. References are provided for events outlined. Phylloidin-stained (left) and anti-phospho-FAK-stained (right) dormant MCF-7 cells on fibronectin are displayed

This study adds further clarity to our in vitro dormancy model of estrogen sensitive human breast cancer cell lines, as illustrated in Fig. 10. It demonstrates that injury to bone marrow stroma can induce the secretion of inflammatory cytokines IL-6 and IL-8 and activate TGFβ1 signaling. These cytokines and growth factor, in turn, can promote dormant ER+ breast cancer cells in our in vitro model to change their phenotype to a mesenchymal appearance and begin to proliferate and migrate. Based on the differences in appearance and behavior between dormant and reawakened cells, the data would suggest that these cytokines and TGFβ1 induced an EMT in these cells. However, our data demonstrated that, surprisingly, the EMT program was already activated in the dormant cells, despite their characteristic appearance as very large epithelioid cells with high cytoplasm to nucleus ratios and round, non-polar shapes with omnidirectional focal complex activation and cortical actin redistribution [34, 37]. Interestingly, ERα was also downregulated in dormant cells or by exogenous cytokines and was further diminished in reactivated dormant cells that acquired the mesenchymal phenotype. This finding may have significant implications in the standard of care use of estrogen binding or synthetic inhibitors in the adjuvant setting of ER+ breast cancer.

EMT events are described as collectively necessary for the acquisition of an invasive phenotype by differentiated epithelial cells in most cases, rendering the cell capable of metastasis. These include an initial dissolution of epithelial intercellular junctions, loss of the characteristic epithelial apical-basal polarity, acquisition of front-rear polarity and reorganization of cytoskeletal architecture with formation of stress fibers [49]. The changes are dependent on the reprogramming of gene expression from a characteristic epithelial expression pattern that includes synthesis of E-cadherin to a characteristic mesenchymal program. The latter includes synthesis of transcription factors Snail1 or Snail2/Slug, ZEB1 or ZEB2 and Twist and a switch to expression of N-cadherin. The mesenchymal program also entails a switch in the integrin repertoire, expression of matrix metalloproteases that enable invasive behavior [49–51] and activation of small GTPase signaling by guanine nucleotide exchange factors (GEFs) responsible for the observed cytoskeletal changes [52, 53]. In other systems, it is well documented that FGF-2 and other ligands of receptor tyrosine kinases induce EMT [54, 55].

In our studies of the dormancy phenotype, we demonstrated that in fact, cells induced into a dormant state by FGF-2 already underwent many of these mesenchymal events, including re-expression of integrins lost with de-differentiation [34] and activation of MAPK [36], p38 [36] and PI3K/protein kinase B (Akt)/Glycogen synthase kinase-3b (GSK3b) [34, 36], Our studies also demonstrated that exogenous FGF-2 induces matrix metalloproteases in breast cancer cells [56]. However, contrary to these events, FGF-2 inhibits proliferation of ER+ breast cancer cells by activation of cell cycle inhibitors p21^WAF1/Cip1^, p27^KIP1^ and p15^INK4B^ resulting in dephosphorylation and activation of retinoblastoma protein (Rb) and cell cycle arrest [35, 48]. Our model demonstrated that dormancy is sustained through omnidirectional activation of focal adhesion complexes, which include Y397-phosphorylation of focal adhesion kinase (FAK) and the inactivation of RhoA through membrane localization of the Rho GAP GRAF [37] (Fig. 10). GRAF binds the C-terminal SH3 domain of the membrane localized FAK [57] and blocks RhoA-mediated stress fiber formation [58], resulting in rearrangement of F-actin to a cortical distribution [37]. These events depend on dual signaling through FGF-2 and fibronectin-mediated outside-in activation of integrin α5β1 [37]. Hence, the activation of the mesenchymal signaling and gene expression pattern in dormant cells is suppressed by the inactivation of RhoA, sustained cortical actin redistribution and focal complex formation, events that collectively maintain the dormant phenotype with a characteristic dormant appearance and impaired motility (Fig. 10). When the dormancy-initiating signaling through FGF-2 and integrin α5β1 is disrupted however, the EMT phenotype becomes evident and the cells take on a mesenchymal appearance, begin to proliferate and become motile. IL-6, IL-8 and TGFβ1 accentuate the proliferative and motile behavior.

Evidence from other systems demonstrates that the inflammatory cytokines IL-6 and IL-8 promote cancer progression. IL-6 and IL-8 can promote the growth of epithelial ovarian cancer cells [59]. IL-8 affects androgen independence [60] and IL-6 induces androgen receptor activation during prostate cancer cell progression [61]. The IL-8-induced androgen-independent growth and migration was shown to be mediated through the oncogene cellular-Src kinase (c-Src) and FAK [62]. These data are not inconsistent with our observations that both IL-6 and IL-8 decrease the expression of ERα in our growing MCF-7 cells. The pro-malignant effects of IL-6 and IL-8 have been reported to be mediated by signaling through several pathways, including through vascular endothelial growth factor (VEGF) [63] through PI3K/Akt [64], by an autocrine loop through epidermal growth factor receptor (EGFR) transactivation mediated by extracellular signal-regulated kinase (ERK) [65], through the gp130 receptor and STAT3 [66, 67], and by p38 MAPK [68]. IL-6 inhibits EGFR promoter methylation, decreasing sensitivity to therapeutic intervention with methylation inhibitors [69]. IL-6 may also induce relative chemotherapy resistance through Akt, myeloid leukemia cell differentiation protein-1 (Mcl-1) and TNF-related apoptosis-inducing ligand (TRAIL) [70–72] and modulation of B-cell lymphoma-2-associated X protein Long (BclXL) and (bcl-2-like protein 4) (BAX), [73]. IL-6 and IL-8 also enable the microenvironment to promote cancer progression by promoting migration and matrix metalloprotease (MMP) production in dermal fibroblasts [74], bone resorption and osteolytic metastases though cyclooxygenase-2 (Cox-2)/prostaglandin E2 (PGE2) [75, 76]. It is evident that these cytokines, as well as growth factors, activate different signal pathways, often depending on whether an epithelial or mesenchymal program is activated in a particular cell.

In contrast to the proliferative effects on dormant cells, rhIL-6 and rhIL-8 did not promote the growing colony forming potential of growing cells. This is also consistent with prior observations where IL-6 undergoes transition from a growth inhibitor associated with neuroendocrine differentiation to a growth stimulator during prostate cancer cell progression [61]. We did observe, that, in fact, rhIL-6, rhIL-8 and rhTGFβ1 decreased the motility of growing epithelial cells (Fig. 3). This effect was associated with and potentially due to the increased expression of E-cadherin by incubation with these factors (Fig. 2).

rhTGFβ1 also had a dual effect on MCF-7 cells, depending on whether they were growing, in which case it inhibited the cells’ potential to form growing colonies (Fig. 1b), or dormant with an activated EMT gene expression program, where it promoted their growing colony forming potential (Fig. 1a). This recapitulated the well-documented opposing effects of TGFβ1 on malignant mammary epithelial cells before and after EMT [77].

The mechanisms of TGFβ1 inhibitory effects on epithelial cells are mediated through activation of cdk inhibitors p16^INK4A^, p15^INK4B^, p21^WAF1/Cip1^, and/or p27^KIP1^ and repression of c-Myc expression through downregulation of the *Inhibitor of Differentiation/DNA binding* 1 (ID1) induced by SMAD 3/4 [78, 79]. Under conditions of intense mitogenic stimulation, TGFβ1 triggers cytostasis or apoptosis, depending on the intensity of the proliferative signal [80, 81]. In this context, we previously demonstrated that FGF-2 activated ERK in MCF-7 cells while simultaneously upregulating p^WAF1/Cip1^ and p27^KIP1^, resulting in G1 cell cycle arrest [82], and downregulated Bcl2 and upregulated BAX resulting in increased apoptosis [83]. In this setting, we previously demonstrated that the growth inhibitory effects of FGF-2 through p21^WAF1/Cip1^ and p27^KIP1^ were mediated through TGFβ1 [48].

The tumor suppressive effects of TGFβ1 are lost in cells that have undergone EMT [49, 78]. Reasons include mutations in transforming growth factor beta receptor II (TGFβRII), discontinuing the induction or deletion of p15^INK4B^, p16^INK4A^ and ARF, promotion of ID1 and induction of c-Myc expression [78], and mutations in SMADs. TGFβ1 promotes EMT by a program dependent on SMAD-mediated transcriptional events that induce SNAIL, SLUG and TWIST [84] as well as SMAD-independent events that promote changes in the cytoskeleton and cell junctional complexes [85] through activation of RhoA [86, 87]. SMADs induce Ras signaling [88] and motility through human epidermal growth factor receptor-2 (Her2/neu) [89]. Signal pathways activated by TGFβ1 in the induction of EMT include the PI3K/Akt/mTOR pathway [49, 90], the ERK pathways [49].

Our data also demonstrate that ER+ breast cancer cells that have undergone activation of the mesenchymal program have a significantly diminished capacity to re-enter dormancy in response to FGF-2. Cells treated with FGF-2 on day 0, disbursed on day 6 and re-incubated with rhFGF-2 form dormant colonies very inefficiently. Parenthetically, we also found that cells treated with TGFβ1 on day 0 also have a decreased capacity to be induced into dormancy by incubation with FGF-2 on day 6 (Fig. 4a). It is clear that a change affects cells during the EMT program that renders them resistant to the ability of FGF-2 to induce cell cycle arrest and the dormancy program previously described [34, 36, 37]. It is possible that EMT may modulate FGF-2 receptor splice variants with mesenchymal transition [91], which may be needed for induction of dormancy. These hypotheses remain the subject of future investigations.

Some insight into the heterogeneity and stochastic nature of recurrence of ER+ breast cancer in the population was gleaned from the lack of reproducibility in the injury data we observed with human stroma. Since we are postulating that injury to stroma over a lifetime either by chemical injury or estrogen deprivation from menopause likely contributes to the random recurrence of dormant micrometastases in the marrow, the capacity of the bone marrow stroma to withstand insult among different patients may very well contribute to the randomness of recurrence. The lack of direct effect of a hypoxic pulse on induction of cytokine secretion was not unexpected. The bone marrow microenvironment is relatively hypoxic [92, 93] and thus cells are adapted to function normally under these conditions. Since aging stroma is most sensitive to injury, a logical corollary would suggest that stroma from young donors would be more resilient. The variable response to injury in stroma from normal volunteers could be explained by age as well as genetic variability. It was the primary reason we decided to conduct our co-cultivation studies with inbred athymic mice. A large number of normal volunteers of variable ages will be needed to investigate this question.

## Conclusions

The main conclusions of the study are:

1. Phenotypically dormant ER+ breast cancer cells in our in vitro dormancy model already have activated EMT gene expression programs and downregulated ERα.

2. Stromal inflammation reactivates the growth and motility of dormant ER+ breast cancer cells and induces a mesenchymal phenotype.

3. Reactivated growing cells feedback activate the secretion of inflammatory cytokines by stroma.

4. Reactivated cells have an impaired ability to re-enter dormancy.

The importance and relevance of these findings relate to the high rate of occurrence of dormant micrometastases in the bone marrow of women with early stage breast cancer. Half of women with ER+ localized breast cancer recur by 10 years after diagnosis, a usually fatal event. It is possible that downregulation of ERα in dormant cells may impact the effects of standard of care adjuvant therapy with estrogen blocking or synthetic inhibitors. These data demonstrate that injury to the bone marrow stroma through oxidation, hypoxia and estrogen deprivation induces reactivation of dormant micrometastases, mediated by stromal inflammatory responses. This establishes a rationale for investigating methods of preventing stromal inflammation as a potential mechanism of suppressing recurrence of micrometastases.

## Abbreviations

Akt: PI3K/protein kinase B
ATCC: American Type Culture Collection
ATF: Activating transcription factor, a cyclic AMP-dependent transcription factor
ATRA: All-*trans* retinoic acid, a retinoic acid isoform
Axl: AXL receptor tyrosine kinase
BAX: Bcl-2-like protein 4
BclXL: B-cell lymphoma-2-associated X protein-long
BMP: Bone morphogenic protein, a member of the transforming growth factor β family
CCCP: Carbonyl-cyanide m-chlorophenylhydrazzone
CDK: Cyclin dependent kinase
Cox-2: Cyclooxygenase-2
c-Src: Cellular c-Src kinase
CXCL1/KC: IL-8
DEC2: A basic helix-loop-helix transcription repressor involved in circadian rhythm
DMEM: Dulbecco’s modifies Eagle’s medium
EGFR: Epidermal growth factor receptor
EMT: Epithelial-mesenchymal transition
ER+: Estrogen receptor positive.
ERα: Estrogen receptor alpha
ERK: Extracellular signal-regulated kinase
F-actin: Fibrillar actin
FAK: Focal adhesion kinase
FBS: Fetal bovine serum
FGF-2: Fibroblast growth factor-2, basic fibroblast growth factor
GAP: Rho GTPase activating protein
GAS6: Growth arrest-specific 6
GEF: Guanine nucleotide exchange factor
GLUT-1: Glucose transporter-1
GRAF: GTPase Regulator Associated with the Focal Adhesion Kinase
GSK3b: Glycogen synthase kinase-3b
GTPase: Guanosine triphosphatase
H_2_O_2_: Hydrogen peroxide
Her2/neu: Human epidermal growth factor receptor-2
HIF1: Hypoxia-inducible factor 1-alpha
HRP: Horseradish preroxidase
HSP70: Heat shock protein 70
HTB-22: Human MCF-7 Breast Adenocarcinoma
ICI 182780: Fulvestrant
ID1: Inhibitor of Differentiation/DNA binding 1
IgG: Immunoglobulin G
IL: Interleukin
LIF: Leukemia inhibitory factor
LOXL2: Lysyl oxidase homolog 2
MAPK: Microtubule-associated protein kinase
MCF-7: Michigan Cancer Foundation-7 breast cancer cells
Mcl-1: Myeloid leukemia cell differentiation protein-1
MMP: Matrix metalloprotease
mTOR: Mammalian target of rapamycin
NANOG: A homeobox transcription factor critically involved with self-renewal of undifferentiated embryonic stem cells
NDRG1: N-myc downregulated gene 1, a member of a protein family which belongs to the alpha/beta hydrolase superfamily
Notch3: Neurogenic locus notch homolog protein 3
NR2F1: Nuclear receptor subfamily 2 group F member 1, an orphan retinoic acid receptor
p21^WAF1^: Cyclin dependent kinase interacting protein 1
p27^Kip1^: Cyclin dependent kinase (CDK) inhibitor p27
p38: A mitogen-activated protein kinase gene activated by stress stimuli
PGE2: Prostaglandin E2
PI3K: Phosphoinositol-3 kinase
RANKL: Receptor activator of nuclear factor kappa-Β ligand
RARβ: Retinoic acid receptor beta
Rb: Retinoblastoma protein
RIPA: Radioimmunoprecipitation assay
rh: Recombinant human
Rheb: Ras homolog enriched in brain GTP-binding protein
RhoA: A member of the small GTPAse proteins regulating stress fibers
SDS-PAGE: Sodium dodecyl sulfate polyacrylamide gel electrophoresis
SLUG: Human embryonic zinc finger transcriptional repressor protein SNAI2 which downregulates expression of E-cadherin
SOCS3: Suppressor of cytokine signaling 3, a member of the STAT-induced STAT inhibitor family
SOX9: A transcription factor of the HMG-box class DNA-binding proteins
STAT3: Signal transducer and activator of transcription protein-3
TGF: Transforming growth factor-β, different members of the family include TGFβ1, TGFβ2 and bone morphogenic proteins (BMPs)
TGFβRII: Transforming growth factor beta receptor II
Tie2: An angiopoietin receptor encoded by the TEK gene
TNF: Tumor necrosis factor alpha
TRAIL: TNF-related apoptosis-inducing ligand
uPA: Urokinase-type plasminogen activator
VCAM-1: Vascular cell adhesion molecule 1
VEGF: Vascular endothelial growth factor
